# Nationwide larval mosquito sampling in Cambodian households: Vector species in anthropogenic breeding sites

**DOI:** 10.1371/journal.pntd.0014342

**Published:** 2026-05-18

**Authors:** Bros Doeurk, Shinji Kasai, Pierre-Olivier Maquart, Sébastien Boyer

**Affiliations:** 1 Medical and Veterinary Entomology Unit, Institut Pasteur du Cambodge, Phnom Penh, Cambodia; 2 Department of Medical Entomology, National Institute of Infectious Diseases, Japan Institute for Health Security, Tokyo, Japan; 3 Institut de Recherche pour le Développement, UMR-247 EGCE IRD, Gif sur Yvette, France; 4 Ecology and Emergence of Arthropod-Borne Diseases, Institut Pasteur, Paris, France; Federal University of Agriculture Abeokuta, NIGERIA

## Abstract

Vector control remains a key strategy in reducing mosquito-borne disease transmission. Understanding mosquito species distribution, diversity, and breeding habitat ecology is crucial for effective surveillance and to define targeted vector control interventions. We conducted a study to understand the diversity and habitat preferences of mosquito larvae across Cambodia during the rainy season from July to September 2024. Mosquito larvae were collected from a variety of breeding habitats located around households across all 25 provinces. The national sampling was conducted once during the rainy season in urban (city) and rural (village) areas within each province. Collected larvae were reared to adult emergence in the insectarium for morphological identification, further confirmed with molecular techniques. We found 37 mosquito species in the households, of which 12 are vectors of pathogens such as dengue and Japanese encephalitis viruses, and *Plasmodium* species, representing 93% of all collected mosquitoes. Larvae were predominantly found in anthropized artificial breeding habitats, accounting for 98% of all larvae collected. Notably, the two primary dengue vectors, *Aedes aegypti* and *Ae. albopictus*, were recorded from all 52 sampling locations. In addition, our study identified the presence of *Aedes vittatus* in 12 provinces, a new confirmed vector of dengue in Cambodia. We also recorded eight Japanese encephalitis vectors, with at least one species from all sampling sites. There were no statistically significant differences in larval mosquito biodiversity (relative abundance, number of species, Shannon and Simpson diversity indices) between cities and villages, with 15 species occurring in both environments, representing 41% of the species and 99% of all mosquitoes collected. The widespread and predominant presence of dengue and Japanese encephalitis vectors in every household confirms the endemic circulation of these diseases in Cambodia.

## Author summary

Mosquito-borne diseases such as dengue, Japanese encephalitis and malaria remain major public health concerns in Cambodia. However, there is limited information on the global distribution of vector species and their breeding habitats. In this study, we collected mosquito larvae across both urban and rural areas in the 25 provinces of Cambodia to assess the diversity of mosquito species related to households. The three main predominant species associated with human inhabitants are well-known vector species, such as *Aedes aegypti*, *Ae. albopictus* and *Culex quinquefasciatus*, and they accounted for 91% of all larvae collected. Notably, the primary dengue vectors *Ae. aegypti* and *Ae. albopictus* were found in all collected locations. Most mosquito larvae were associated with anthropogenic artificial containers, indicating a strong preference of vector species to human-modified environments. These results provide critical ecological insights to guide targeted and sustainable vector surveillance and control strategies in Cambodia, especially under ongoing environmental and urban transformations.

## Introduction

Mosquitoes are known to be vectors of numerous pathogens, playing a crucial role in the transmission of vector-borne diseases globally [1,2]. Recently, the problem of mosquito-borne infections has spread to areas where it was not previously recorded, driven by the widespread distribution of mosquito vectors [3,4]. In Cambodia, a total of 312 mosquito species have been recorded, with 43 species recognized for their medical importance [5]. Among them, the three most common genera in the county are *Aedes*, *Anopheles* and *Culex*, which are of particular concern due to their association with disease transmission such as dengue fever, chikungunya, Zika, malaria and Japanese encephalitis [6–9].

Dengue is currently the most important and widespread arboviral disease in Cambodia, with thousands of cases reported annually throughout the country [10]. In Cambodia, all four dengue virus serotypes (DENV-1 to DENV-4) are endemic, with DENV-1 and DENV-2 reported as the predominant serotypes in both past (2012) and recent (2019) outbreaks [10]. A recent study highlighted the widespread distribution of *Ae. aegypti* and *Ae. albopictus*, the two primary dengue vectors, across all 25 provinces in Cambodia and shows that these species have adapted to diverse ecological settings [6]. The widespread presence of these main dengue vectors poses a significant challenge to reducing dengue outbreaks through vector control efforts in the country [11], especially as resistance to all commonly used insecticides has been reported among local populations [12–14]. The immature stages of the two species develop in water, within a wide range of larval habitats from natural, such as tree holes and ground pools, anthropized natural, such as coconut shells, ditches and rice fields, to artificial habitats, such as concrete, plastic and polystyrene containers, discarded tyres, jars and buckets [6]. Both species are predominantly found in anthropized artificial containers and are strongly influenced by human activities [6].

In Cambodia, Japanese encephalitis virus (JEV) continues to pose a serious threat to public health, especially for children living in rural areas [8,15]. The significance of animal reservoirs in maintaining the transmission cycle was confirmed by a multi-host serological study conducted in Kandal province, which revealed widespread JEV circulation among animals, with 31% of pigs, 12% of ducks, and 35% of dogs carrying antibodies [16]. In Cambodia, the JEV was first isolated from *Culex tritaeniorhynchus* mosquitoes in 1965 [17]. The presence of *Culex* mosquito vectors is closely linked to JEV transmission. In rural areas, these vectors usually breed in rice paddies and other flooded habitats [17–19].

Malaria remains a significant vector-borne disease in forested areas in Cambodia, although its impact has decreased markedly over the past two decades: between 2010 and 2020, a reduction of over 90% malaria cases was observed, due to improved access to diagnosis, effective treatment and vector control measures [20]. Transmission is primarily only maintained by *Anopheles* mosquitoes, with *An. baimaii*, *An. dirus*, *An. maculatus* and *An. minimus* serving as the main vectors, especially in forested and forest-fringe regions where malaria remains endemic [7,21].

Overall, larval habitats of mosquitoes in Cambodia remain poorly understood, especially in household environment. Indeed, breeding habitats of dengue vectors in Cambodia have been summarized in a review based on a sampling conducted in only one province [6], while only one study investigated the breeding habitats of malaria vectors in forest areas [22]. For other mosquito species, including JEV vectors, the checklist detailed many breeding habitats but is solely based on the literature [2]. Since mosquito-borne disease prevention still relies on controlling mosquito populations, there is an urgent need to study vector distribution and ecological characteristics of their breeding habitats. This study aims to describe the larval breeding sites of mosquitoes in household compounds in both city and village areas across Cambodia.

## Methods

### Ethics statement

This study did not require ethical approval as this study did not involve human or animal participants. The individual in this photograph has given written informed consent (as outlined in PLOS consent form) to publish this image.

### Sample collection

**Fig 1:**
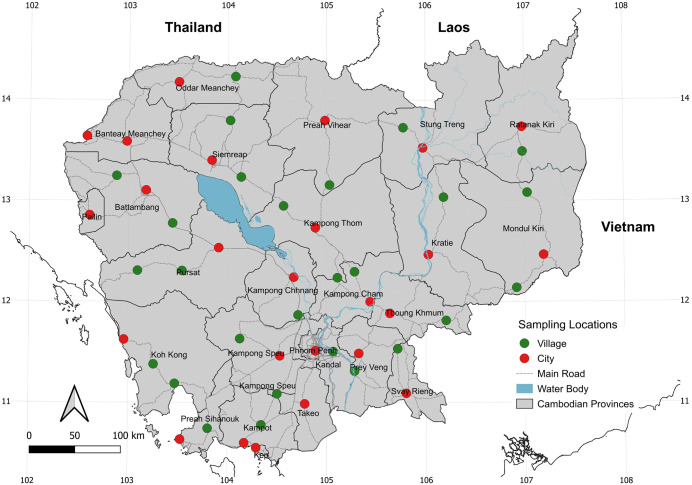
Map of Cambodia showing 52 mosquito larval sampling locations across 25 provinces. Maps were generated using the free, open-source QGIS software. Subnational administrative boundary shapefiles for Cambodia and neighboring countries are available for download from The Humanitarian Data Exchange (https://data.humdata.org/dataset/).

The study was conducted at 52 locations along the national roads across all 25 provinces of Cambodia (Fig 1). Of these, 25 sites were in urban areas (cities) and 27 in rural areas (villages), with generally one city and one village selected from each province, except some provinces where one or three locations were sampled depending on the size of the province. The sampling focused on anthropogenic breeding habitats, including domestic and peri-domestic containers, located in household compounds. Indeed, human-dominated environments represent key ecological breeding habitats for main mosquito vector species. and are relevant for community and national-level vector control strategies. During the collection, larvae and pupae were searched for and collected from a variety of breeding habitats found around households. Each location was collected once during the rainy season (July-September 2024), a period known for diverse mosquito breeding habitats [6]. At each site, larval collections were conducted in approximately 10 households, with a maximum sampling duration of around one hour and a maximum of two hours per site. Surveys were carried out by a team of four technicians following a designed larval sampling and data-recording protocol: all breeding habitats were emptied, and all larvae and pupae present were collected using dippers and larvae nets. The immature stages were then transported to the insectarium at the Institut Pasteur du Cambodge for rearing to adult emergence. Adult mosquitoes were identified morphologically to the species level using a specific taxonomic key [23,24]. In addition to morphological identification, COI sequencing was used to confirm species identification by randomly selecting individuals from each species [25].

### Data analysis

Data analysis focused on exploring the composition and diversity of mosquito species across breeding habitats in both city and village settings. We calculated standard ecological indices to assess mosquito diversity such as relative abundance (total number of individuals per species), species richness (total number of species), Shannon diversity index, which accounts for both abundance and evenness of species, and Simpson’s diversity index, which emphasizes the dominance of species within provinces. To compare the mosquito diversity and relative abundance of each mosquito species between city and village, independent *t*-tests were applied where assumptions of normality were met, as assessed using the Shapiro-Wilk test. Moreover, Jaccard similarity index was used to quantify species composition similarity between city and village. This index reflects the proportion of shared species relative to the total species observed in both landscapes (city and village). All statistical analyses were performed using R software version 4.4.2 [26]. Data analysis was conducted using the ‘tidyverse’ package. Ecological diversity indices and similarity measures, including Shannon, Simpson and Jaccard indices, were calculated using the ‘vegan’ package, and figure visualization was using ‘ggplot2’ packages. The statistical significance was accepted at a *P*-value < 0.05.

## Results

A total of 40,811 mosquitoes were collected from 52 locations across Cambodia. Based on morphological identification, 40,458 individuals (99% of the total collection) were identified to the species level, while 353 individuals (approximately 1%) were identified only to the genus level. A total of 37 mosquito species belonging to 8 genera were found in the study. Among them, 82% mosquitoes (33,386 individuals) were *Aedes*, 16% (6,570) were *Culex*, and the remaining 2% included *Anopheles*, *Armigeres*, *Lutzia*, *Mimomyia*, *Toxorhynchites*, and *Uranotaenia* genera. Twelve species are known as vectors of pathogens, representing for more than 93% (37,465 individuals) of all identified mosquitoes (S1 Table). Three vector species were the most found, with *Ae. aegypti* being the predominant species, accounting for 57% (23,059 individuals) of mosquitoes collected, followed by *Ae. albopictus* for 25% (9,946) and *Cx. quinquefasciatus* for 9% (3,721) (Tables 1 and S1).

**Table 1:** Total number of larval mosquitoes collected across Cambodia by species and breeding habitats. Anthropized artificial habitats include concrete containers, flower pots, glass containers, metal containers, and discarded tires. Anthropized natural habitats include coconut shells. Natural habitats include ground pools and tree holes. Mosquito species marked with an asterisk (*) indicate known disease vectors. (N) and (%) represent the total number of larval mosquitoes collected per species within each habitat type. Total (N) and (%) correspond to the overall abundance and percentage of each species among all collected mosquitoes.

Recorded species	Mosquitoes collected in anthropized artificial habitats		Mosquitoes collected in anthropized natural habitats		Mosquitoes collected in natural habitats		Total number of mosquitoes collected in each species	
	(N)	(%)	(N)	(%)	(N)	(%)	(N)	(%)
Aedes aegypti*	22,883	99	63	0	113	0	23,059	57
*Aedes albolinaetus*	1	100	0	0	0	0	1	0
Aedes albopictus*	9,598	97	247	2	101	1	9,946	25
*Aedes amesii*	6	15	34	85	0	0	40	0
*Aedes gardnerii*	6	100	0	0	0	0	6	0
*Aedes macfarlanei*	13	100	0	0	0	0	13	0
*Aedes prominens*	5	100	0	0	0	0	5	0
*Aedes saxicola*	1	100	0	0	0	0	1	0
Aedes vittatus*	246	98	5	2	0	0	251	1
*Aedes w.albus*	1	100	0	0	0	0	1	0
Anopheles aconitus*	4	100	0	0	0	0	4	0
*Anopheles indefinitus*	23	100	0	0	0	0	23	0
*Anopheles separatus*	2	100	0	0	0	0	2	0
Anopheles vagus*	20	100	0	0	0	0	20	0
*Armigeres kesseli*	26	19	113	81	0	0	139	0
*Armigeres malayi*	0	0	11	100	0	0	11	0
Armigeres subalbatus*	178	76	57	24	0	0	235	1
*Armigeres theobaldi*	10	14	64	86	0	0	74	0
*Culex brevipalpis*	2,435	99	8	0	9	0	2,452	6
Culex fuscocephala*	59	100	0	0	0	0	59	0
Culex gelidus*	63	100	0	0	0	0	63	0
*Culex infantulus*	2	100	0	0	0	0	2	0
*Culex mimulus complex*	12	100	0	0	0	0	12	0
*Culex nigropunctatus*	96	93	0	0	7	7	103	0
Culex pseudovishnui*	6	100	0	0	0	0	6	0
Culex quinquefasciatus*	3,698	99	11	0	12	0	3,721	9
*Culex sinensis*	2	100	0	0	0	0	2	0
Culex tritaeniorhynchus*	2	100	0	0	0	0	2	0
*Culex vishnui.*g*	98	99	1	1	0	0	99	0
*Culex whitmorei*	1	100	0	0	0	0	1	0
*Culex wilfredi.*g	7	100	0	0	0	0	7	0
*Lutzia fuscana*	63	100	0	0	0	0	63	0
*Lutzia vorax*	16	100	0	0	0	0	16	0
*Mimomyia luzonensis*	12	100	0	0	0	0	12	0
*Toxorhynchites splendens*	1	100	0	0	0	0	1	0
*Uranotaenia abdita*	4	100	0	0	0	0	4	0
*Uranotaenia demeilloni*	2	100	0	0	0	0	2	0
Grand Total	39,602	98	614	2	242	1	40,458	

**Fig 2:**
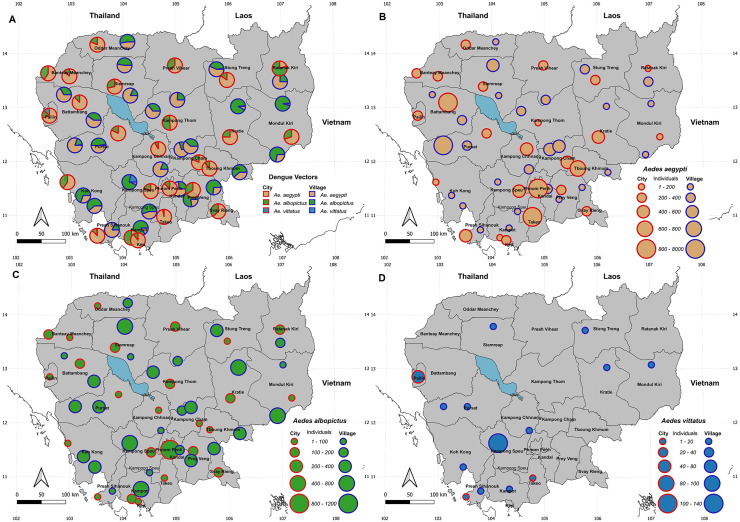
Map showing the distribution and relative density of all dengue vector species and of each recorded species across 25 provinces in Cambodia. Maps were generated using the free, open-source QGIS software. Subnational administrative boundary shapefiles for Cambodia and neighboring countries are available for download from The Humanitarian Data Exchange (https://data.humdata.org/dataset/).

**Fig 3:**
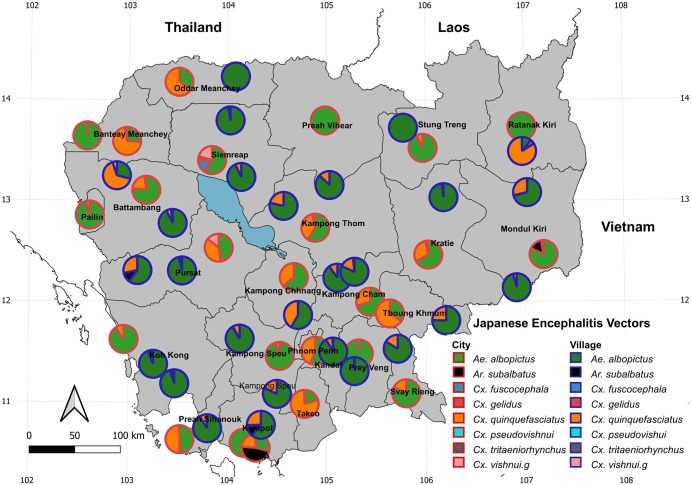
Map showing the distribution and proportion of Japanese encephalitis vectors recorded across 25 provinces in Cambodia. Maps were generated using the free, open-source QGIS software. Subnational administrative boundary shapefiles for Cambodia and neighboring countries are available for download from The Humanitarian Data Exchange (https://data.humdata.org/dataset/).

**Fig 4:**
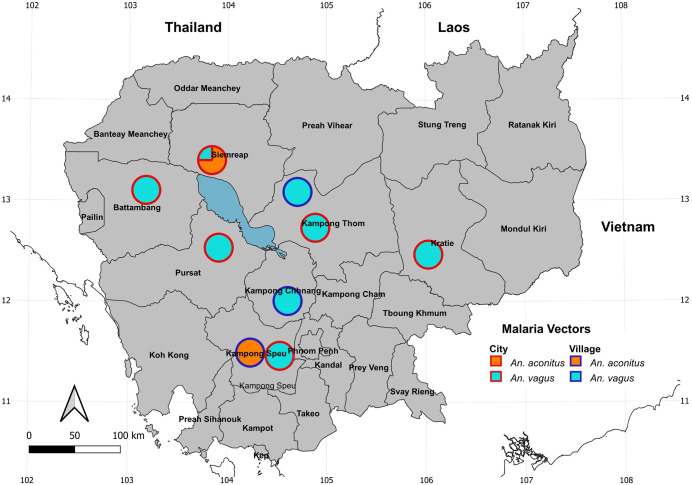
Map showing the distribution and proportion of malaria vectors recorded across 25 provinces in Cambodia. Maps were generated using the free, open-source QGIS software. Subnational administrative boundary shapefiles for Cambodia and neighboring countries are available for download from The Humanitarian Data Exchange (https://data.humdata.org/dataset/).

Larvae were recorded from three main types of breeding habitats: anthropized artificial habitats (98%; concrete, flower pot, glass, metal, plastic, polystyrene and discarded tyre), anthropized natural (2%; coconut shell), and natural (<1%; ground pool and tree hole) (Tables 1 and S1). We recorded three mosquito species known to be involved in dengue transmission: *Ae. aegypti* and *Ae. albopictus*, as primary vectors, and *Ae. vittatus*, as secondary vector. *Aedes aegypti* and *Ae. albopictus* were found across all sampling locations in cities and villages (57% and 25% of all identified mosquitoes, respectively) and in all types of breeding habitats, with a predominance in anthropized breeding habitats (99% and 97%, respectively) (Table 1 and Fig 2). *Aedes vittatus* was recorded in 14 locations across 12 provinces (Kampong Cham, Kampong Speu, Kampot, Koh Kong, Kratie, Mondulkiri, Pailin, Preah Sihanouk, Pursat, Siem Reap, Stung Treng, and Takeo), with 11 of these locations recorded in village areas (Fig 2). Eight mosquito species known to be vectors of Japanese encephalitis (JE) were collected across all 25 provinces. Among them, *Ae. albopictus* was the most widespread (52 locations), followed by *Cx. quinquefasciatus* (46 locations), *Cx. vishnui.*g (15), *Ar. subalbatus* (9), *Cx. fuscocephala* (5), *Cx. gelidus* (2), *Cx. tritaeniorhynchus* (1), and *Cx. pseudovishnui* (1) (Figs 3 and S1). Approximately 60% of these JE vector occurrences were recorded in villages and were associated with anthropized breeding habitats (97%) (Tables 1 and S1). Additionally, we recorded two *Anopheles* species known to be vectors of *Plasmodium*: *An. vagus* (8 locations) and *An. aconitus* (2 locations) (Fig 4). Notably, the two vector species were collected exclusively in anthropized breeding habitats, such as concrete, plastic and metal containers and discarded tyres (Tables 1 and S1).

**Fig 5:**
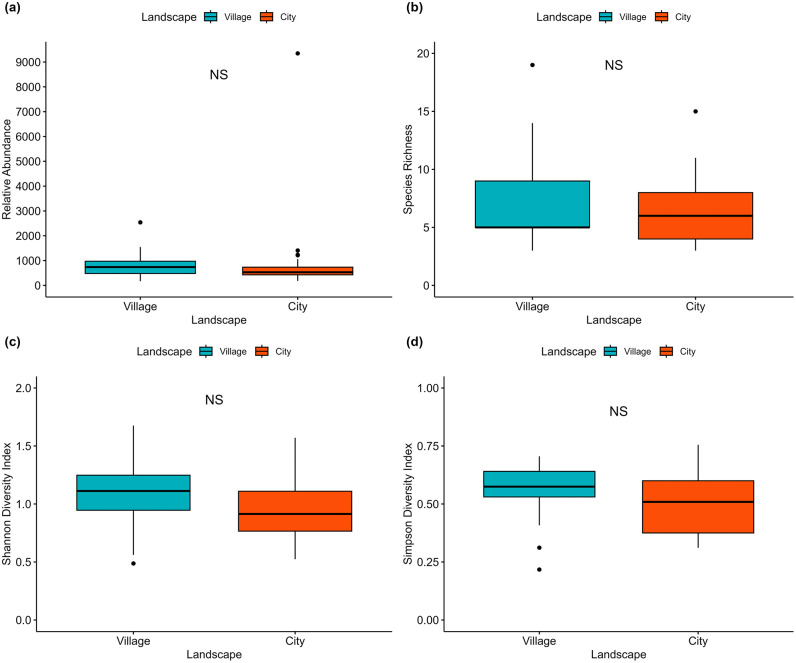
Boxplot showing the comparison of mosquito species diversity between city and village landscapes across Cambodia.

**Fig 6:**
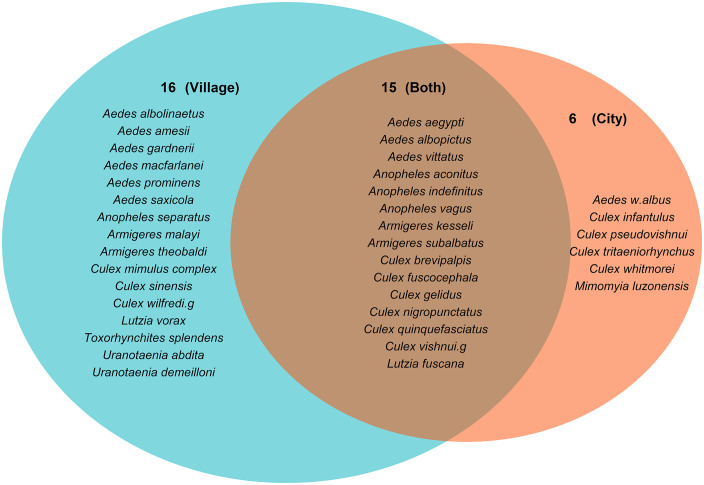
Venn diagram showing Jaccard similarity index of mosquito species composition between city and village.

On average, we recorded 980 ± 370 mosquitoes in cities and 819 ± 118 in villages. The mean species richness was 6.38 ± 0.58 species in city areas and 7.14 ± 0.89 in village areas. The Shannon diversity index (H′) was 0.96 ± 0.06 and 1.07 ± 0.06, and the Simpson diversity index (D) was 0.50 ± 0.03 and 0.56 ± 0.03 in city and village areas, respectively. However, no significant variation in relative abundance, species richness, and Shannon and Simpson diversity indices between city and village landscapes has been observed (Fig 5). Among the 37 identified species, 15 were found in both city and village household compounds, representing 41% of the total species collected and more than 99% of the total abundance. Additionally, 16 species (43%) were recorded exclusively in villages, while 6 species (16%) were recorded only in cities (Fig 6). The Jaccard index between cities and villages was 0.41, indicating a low similarity in mosquito species composition between the two environments. Interestingly, *Ae. albopictus* was the only species found in significantly higher abundance in villages compared to cities (*t*-test, P = 0.015), while other mosquito species showed no significant differences between the two landscapes (*t*-test, P > 0.05, S2 Fig).

## Discussion

Our study highlighted the predominance of *Ae. aegypti* and *Ae. albopictus* in households throughout Cambodia, both species being primary dengue vectors [6]. Both species were found in all types of breeding habitats recorded in this study, indicating their ecological adaptability and capacity to use a wide range of breeding sites, from artificial containers to natural habitats [25]. These two species are well known to be widely and abundantly distributed in tropical and sub-tropical countries [6,27]. Therefore, founding them everywhere during the rainy season is not surprising. In addition, we recorded the presence of *Ae. vittatus*, which is another confirmed dengue vector in Cambodia. Indeed, *Ae. vittatus* has been reported as naturally infected with DENV-2 in Senegal [28], and it has been demonstrated experimentally that it was able to transmit Dengue viruses [29]. Given its wide distribution across many provinces and presence in both village and city landscapes, *Ae. vittatus* may be another important vector of dengue virus transmission in Cambodia, and its vector competence should be tested to assess its role in dengue circulation locally.

Japanese encephalitis (JE) is one of the major mosquito-borne arboviruses circulating in Southeast Asia, including in Cambodia [30]. So far, 14 mosquito species have been confirmed as vectors in Cambodia, 11 considered as potential vectors based on experimental evidence, and 26 suspected vectors based on field virus isolations [31]. In households, we recorded eight species of JEV vectors. At least two JEV vector species was recorded from all 25 provinces, both villages and cities and all recorded breeding habitats in Cambodia. The predominance of these species in villages (60%) is consistent with previous studies showing that JEV vectors are primarily found in rural areas, but can also occur in suburban environments [8,27], while majority of the cities cannot really been considered as totally urbanized. Most JEV vectors belong to the genus *Culex* spp., with a marked zoophilic preference for pigs, the principal amplifying hosts [8,30]. From these vectors, the 99 individuals identified as *Culex vishnui* complex are known to be difficult to separate morphologically [25]. We acknowledge possible uncertainty in their identification and recommend combining morphological and molecular approaches in future studies for more accurate species determination. The breeding habitats of JE vector species are mainly as natural habitats such as rice fields, puddles, ditches, small stream [2]. In our study, we specifically recorded the breeding of each species in a variety of artificial and natural containers, including concrete, flower pots, glass, metal, polystyrene containers, tires, coconut shells, ground pools, and tree holes. In addition to their known natural breeding habitats, we have now recorded these species in a wide range of artificial breeding containers, indicating their adaptability to human modified environments. These findings provide a more comprehensive understanding of the breeding habitat ecology of JEV vectors in Cambodia. Finally, the widespread presence and predominant of *Ae. albopictus* and *Cx. quinquefasciatus* across all 25 provinces in Cambodia suggests a potential high risk for JE virus transmission in the country.

Two *Anopheles* species, *An. aconitus* and *An. vagus*, known as secondary vectors of malaria in Cambodia, were also sampled in households [7,21]. Although these two species were not predominant in our study (only 24 individuals collected), they have been screened to carry *Plasmodium* spp. through natural infection in forested areas of Cambodia [7,21]. Generally, the common breeding habitats of malaria vectors are natural sites including shaded streams, temporary pools, rice fields, slow-moving waters, jungle pools and animal footprints [32,33]. However, their breeding sites in our study were exclusively anthropized breeding habitats, suggesting behavioral plasticity and potential adaptation to human environments. Even if this behavior to exploit artificial containers as larval habitats has been already observed in urban and peri-urban settings in Africa, South America, and Asia [34,35], this is the first observations of malaria vector breeding habitats in Cambodia. This may have important implications for malaria transmission dynamics in areas undergoing rapid land use change. Our study also highlighted a knowledge gap regarding the breeding habitats of *Anopheles* species in Cambodia, which could be more precisely characterized for each species.

Mosquito species diversity was evaluated using the relative abundance, species richness, Shannon and Simpson diversity indices which statistical comparisons showed no significant differences between city and village landscapes. Our findings contrast from other reports, where mosquito abundance and species richness are typically higher in natural and rural areas compared to urban ones [36–38]. These findings demonstrated that natural environments support greater mosquito biodiversity, while urban areas show lower diversity due to the higher dominance for some particular species [39,40]. However, the similarity in diversity observed in our study may be attributable to the comparable anthropogenic breeding habitats sampled across both environments, as our survey primarily focused on human households. Indeed, although the surrounding environments of households may differ (e.g., city and village settings), similar anthropogenic features are consistently observed across households, including comparable breeding sites such as water storage jars, plastic containers, road proximity, and open areas surrounding houses. In addition, the nationwide sampling covered 25 provinces across Cambodia, with similar climatic conditions during the sampling period, rainy and hot, leading to similar ecological and environmental characteristics. In addition, diversity indices such as Shannon and Simpson incorporate not only species presence across landscapes but also their relative abundances, which may reduce apparent differences in diversity despite variations in species composition, as more than 99.5% of individuals were shared between landscapes.

This bring the limitation of our approach is the exclusive focus on anthropized habitats near human settlements, which may underestimate the presence of species preferring more natural or less disturbed environments, particularly in natural areas. Our nationwide sampling was designed primarily to target the 2 main dengue vector species and our results emphasize species presence near humans rather than their relative abundance or density. In addition, our larval sampling was conducted only once during the rainy season, when mosquito diversity and breeding activity are expected to be highest [6]. Therefore, our results provide a snapshot of mosquito species distribution and may not reflect seasonal variation throughout the year.

Our findings show that the most productive breeding habitats in household compounds were plastic and concrete containers, and all major vector species preferentially breed in these containers. This finding aligns with a report conducted in Cambodia across different landscapes, including urban, residential, river, wooded, wetland, and rice field areas in Kampong Thom province [6]. These habitats should therefore prioritized for targeted interventions to effectively reduce mosquito populations and lower the risk of mosquito-borne disease transmission in the country. These findings emphasize the need for targeted container-based control strategies, including container removal and proper management, as well as improved water storage practices. In addition, mapping the distribution of vector species is essential for strengthening national surveillance systems.

## Conclusion

Our study provides a more complete overview, identifying species in anthropized artificial, anthropized natural, and natural habitats of other vectors, highlighting their ecological adaptability and the need for broader surveillance. We recorded the widespread distribution and predominance of three key mosquito species *Ae. aegypti*, *Ae. albopictus*, and *Cx. quinquefasciatus* across Cambodia. These three species play a significant role in pathogen transmission to humans such as dengue and Japanese encephalitis, as they are predominantly associated with anthropized habitats, in particular near human households. Their ecological adaptability highlights the need to consider a wide range of habitat types and landscapes in vector surveillance and control programs, particularly at a time of ongoing urbanization and environmental change. Future efforts should include comprehensive, longitudinal and systematic sampling to better characterize vector breeding habitats, especially in underexplored and natural environments.

## Supporting information

S1 Fig Map showing the distribution and relative abundance of each Japanese encephalitis vector recorded across 25 provinces in Cambodia. Maps were generated using the free, open-source QGIS software. Subnational administrative boundary shapefiles for Cambodia and neighboring countries are available for download from The Humanitarian Data Exchange (https://data.humdata.org/dataset/).
(PNG)

S2 Fig Average number of mosquitoes collected per province in urban (city) and rural (village) areas. Bar plots represent mean ± standard error. Asterisks indicate statistically significant differences between landscapes.
(TIF)

S3 Fig Graphical abstract. Maps were generated using the free, open-source QGIS software. Subnational administrative boundary shapefiles for Cambodia and neighboring countries are available for download from The Humanitarian Data Exchange (https://data.humdata.org/dataset/).The individual in this photograph has given written informed consent (as outlined in PLOS consent form) to publish this image.
(TIF)

S1 Table Number of mosquito larvae collected from different landscape (village and city) and different breeding habitats across Cambodia. (DOCX)
