# Supplementary figures and images for: Nationwide larval mosquito sampling in Cambodian households: Vector species in anthropogenic breeding sites
Source: PLoS Negl Trop Dis. 2026 May 18;20(5):e0014342. doi: 10.1371/journal.pntd.0014342 (PMC13197075; doi:10.1371/journal.pntd.0014342)

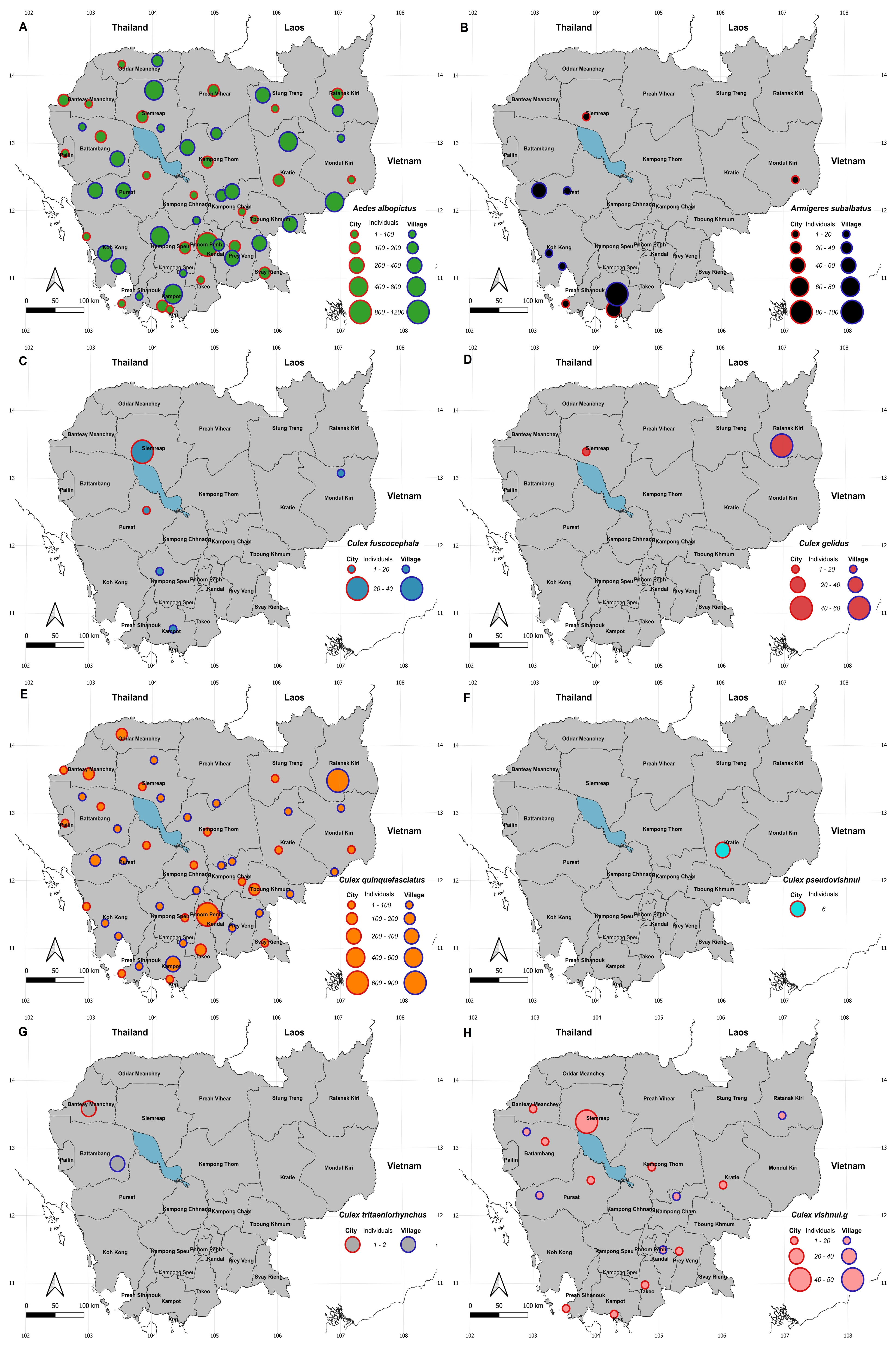

Supplement: S1 Fig — Maps were generated using the free, open-source QGIS software. Subnational administrative boundary shapefiles for Cambodia and neighboring countries are available for download from The Humanitarian Data Exchange (https://data.humdata.org/dataset/). (PNG) [file pntd.0014342.s001.png]
