# Supplementary material for: Nationwide larval mosquito sampling in Cambodian households: Vector species in anthropogenic breeding sites
Source: PLoS Negl Trop Dis. 2026 May 18;20(5):e0014342. doi: 10.1371/journal.pntd.0014342 (PMC13197075; doi:10.1371/journal.pntd.0014342)
Supplement: S1 Table — (DOCX) [file pntd.0014342.s004.docx]

| Species | Vector status | Village (%) | | City (%) | | Concrete container | Flower pot | Glass | Metal container | Plastic container | Polystyrene container | Tyre | Coconut shell | Ground pool | Tree hole | Grand Total | Grand Total (%) |
| --- | --- | --- | --- | --- | --- | --- | --- | --- | --- | --- | --- | --- | --- | --- | --- | --- | --- |
| *Aedes aegypti* | DENV, ZIKV, CHYKV, RVFV, WNV, YFV | 6,608 | 29% | 16,385 | 71% | 5,599 | 2,340 | 34 | 1,278 | 8,652 | 273 | 4,707 | 63 | 29 | 84 | 23,059 | 57% |
| *Aedes albolinaetus* |  | 1 | 100% | 0 | 0% | 0 | 0 | 0 | 1 | 0 | 0 | 0 | 0 | 0 | 0 | 1 | 0% |
| *Aedes albopictus* | DENV, ZIKV, CHYKV, JEV, RVFV, WNV, YFV | 6,327 | 64% | 3,592 | 36% | 1,825 | 48 | 28 | 657 | 4,497 | 342 | 2,201 | 247 | 2 | 99 | 9,946 | 25% |
| *Aedes amesii* |  | 40 | 100% | 0 | 0% | 0 | 0 | 0 | 0 | 0 | 0 | 6 | 34 | 0 | 0 | 40 | 0% |
| *Aedes gardnerii* |  | 6 | 100% | 0 | 0% | 0 | 0 | 0 | 0 | 0 | 0 | 6 | 0 | 0 | 0 | 6 | 0% |
| *Aedes macfarlanei* |  | 13 | 100% | 0 | 0% | 10 | 0 | 0 | 0 | 0 | 0 | 3 | 0 | 0 | 0 | 13 | 0% |
| *Aedes prominens* |  | 5 | 100% | 0 | 0% | 3 | 0 | 0 | 0 | 1 | 0 | 1 | 0 | 0 | 0 | 5 | 0% |
| *Aedes saxicola* |  | 1 | 100% | 0 | 0% | 1 | 0 | 0 | 0 | 0 | 0 | 0 | 0 | 0 | 0 | 1 | 0% |
| *Aedes vittatus* | YFV, DENV, CHIKV | 195 | 78% | 56 | 22% | 190 | 0 | 0 | 16 | 26 | 0 | 14 | 5 | 0 | 0 | 251 | 1% |
| *Aedes w.albus* |  | 0 | 0% | 1 | 100% | 0 | 0 | 0 | 0 | 1 | 0 | 0 | 0 | 0 | 0 | 1 | 0% |
| *Anopheles aconitus* | MAL | 1 | 25% | 3 | 75% | 0 | 0 | 0 | 0 | 3 | 0 | 1 | 0 | 0 | 0 | 4 | 0% |
| *Anopheles indefinitus* |  | 3 | 13% | 20 | 87% | 18 | 0 | 0 | 5 | 0 | 0 | 0 | 0 | 0 | 0 | 23 | 0% |
| *Anopheles separatus* |  | 2 | 100% | 0 | 0% | 2 | 0 | 0 | 0 | 0 | 0 | 0 | 0 | 0 | 0 | 2 | 0% |
| *Anopheles vagus* | MAL | 6 | 30% | 14 | 70% | 10 | 0 | 0 | 3 | 4 | 0 | 3 | 0 | 0 | 0 | 20 | 0% |
| *Armigeres kesseli* |  | 138 | 99% | 1 | 1% | 10 | 0 | 0 | 0 | 7 | 0 | 9 | 113 | 0 | 0 | 139 | 0% |
| *Armigeres malayi* |  | 11 | 100% | 0 | 0% | 0 | 0 | 0 | 0 | 0 | 0 | 0 | 11 | 0 | 0 | 11 | 0% |
| *Armigeres subalbatus* | JEV, FILWB | 160 | 68% | 75 | 32% | 54 | 0 | 0 | 94 | 14 | 0 | 16 | 57 | 0 | 0 | 235 | 1% |
| *Armigeres theobaldi* |  | 74 | 100% | 0 | 0% | 2 | 0 | 0 | 0 | 8 | 0 | 0 | 64 | 0 | 0 | 74 | 0% |
| *Culex brevipalpis* |  | 1,335 | 54% | 1117 | 46% | 509 | 0 | 0 | 239 | 1,042 | 0 | 645 | 8 | 0 | 9 | 2,452 | 6% |
| *Culex fuscocephala* | JEV | 24 | 41% | 35 | 59% | 48 | 0 | 0 | 0 | 10 | 0 | 1 | 0 | 0 | 0 | 59 | 0% |
| *Culex gelidus* | JEV | 54 | 86% | 9 | 14% | 4 | 0 | 0 | 0 | 59 | 0 | 0 | 0 | 0 | 0 | 63 | 0% |
| *Culex infantulus* |  | 0 | 0% | 2 | 100% | 0 | 0 | 0 | 1 | 1 | 0 | 0 | 0 | 0 | 0 | 2 | 0% |
| *Culex mimulus complex* |  | 12 | 100% | 0 | 0% | 12 | 0 | 0 | 0 | 0 | 0 | 0 | 0 | 0 | 0 | 12 | 0% |
| *Culex nigropunctatus* |  | 16 | 16% | 87 | 84% | 0 | 0 | 0 | 1 | 95 | 0 | 0 | 0 | 0 | 7 | 103 | 0% |
| *Culex pseudovishnui* | JEV | 0 | 0% | 6 | 100% | 0 | 0 | 0 | 0 | 0 | 0 | 6 | 0 | 0 | 0 | 6 | 0% |
| *Culex quinquefasciatus* | JEV | 1,799 | 48% | 1,921 | 52% | 929 | 540 | 0 | 116 | 1,463 | 0 | 650 | 11 | 0 | 12 | 3,721 | 9% |
| *Culex sinensis* |  | 2 | 100% | 0 | 0% | 0 | 0 | 0 | 2 | 0 | 0 | 0 | 0 | 0 | 0 | 2 | 0% |
| *Culex tritaeniorhynchus* | JEV, RVFV | 0 | 0% | 2 | 100% | 2 | 0 | 0 | 0 | 0 | 0 | 0 | 0 | 0 | 0 | 2 | 0% |
| *Culex vishnui.g* | JEV | 17 | 17% | 82 | 83% | 20 | 0 | 1 | 6 | 43 | 0 | 28 | 1 | 0 | 0 | 99 | 0% |
| *Culex whitmorei* |  | 0 | 0% | 1 | 100% | 0 | 0 | 0 | 0 | 1 | 0 | 0 | 0 | 0 | 0 | 1 | 0% |
| *Culex wilfredi.g* |  | 7 | 100% | 0 | 0% | 0 | 0 | 0 | 0 | 4 | 0 | 3 | 0 | 0 | 0 | 7 | 0% |
| *Lutzia fuscana* |  | 33 | 52% | 30 | 48% | 28 | 0 | 0 | 5 | 9 | 0 | 21 | 0 | 0 | 0 | 63 | 0% |
| *Lutzia vorax* |  | 16 | 100% | 0 | 0% | 1 | 0 | 0 | 0 | 1 | 0 | 14 | 0 | 0 | 0 | 16 | 0% |
| *Mimomyia luzonensis* |  | 0 | 0% | 12 | 100% | 1 | 0 | 0 | 0 | 11 | 0 | 0 | 0 | 0 | 0 | 12 | 0% |
| *Toxorhynchites splendens* |  | 1 | 100% | 0 | 0% | 0 | 0 | 0 | 0 | 0 | 0 | 0 | 0 | 0 | 0 | 1 | 0% |
| *Uranotaenia abdita* |  | 4 | 100% | 0 | 0% | 0 | 0 | 0 | 0 | 0 | 0 | 4 | 0 | 0 | 0 | 4 | 0% |
| *Uranotaenia demeilloni* |  | 2 | 100% | 0 | 0% | 0 | 0 | 0 | 0 | 0 | 0 | 2 | 0 | 0 | 0 | 2 | 0% |
| Grand Total |  | 16,913 |  | 23,451 |  | 9,278 | 2,928 | 63 | 2,424 | 15,952 | 615 | 8,341 | 614 | 31 | 211 | 40,458 |  |
| Grand Total (%) |  | 42% |  | 58% |  | 23% | 7% | 0% | 6% | 39% | 2% | 21% | 2% | 0% | 1% |  |  |
| Vectors |  | 15,197 |  | 22,194 |  | 8,681 | 2,928 | 63 | 2,170 | 14,771 | 615 | 7,627 | 384 | 31 | 195 | 37,465 |  |
| Vectors (%) |  | 41% |  | 59% |  | 21% | 7% | 0% | 5% | 37% | 2% | 19% | 1% | 0% | 0% | 93% |  |

S1 Table. Number of mosquito larvae collected from different landscape (village and city) and different breeding habitats across Cambodia
